# Current Continuing Professional Education Practice among Malaysian Nurses

**DOI:** 10.1155/2014/126748

**Published:** 2014-01-09

**Authors:** Mei Chan Chong, Karen Francis, Simon Cooper, Khatijah Lim Abdullah

**Affiliations:** ^1^Department of Nursing Science, Faculty of Medicine, University of Malaya, 50603 Kuala Lumpur, Malaysia; ^2^Charles Sturt University, Wagga Wagga, NSW 2678, Australia; ^3^Monash University, Berwick, Vic. 3806, Australia

## Abstract

Nurses need to participate in CPE to update their knowledge and increase their competencies. This research was carried out to explore their current practice and the future general needs for CPE. This cross-sectional descriptive study involved registered nurses from government hospitals and health clinics from Peninsular Malaysia. Multistage cluster sampling was used to recruit 1000 nurses from four states of Malaysia. Self-explanatory questionnaires were used to collect the data, which were analyzed using SPSS version 16. Seven hundred and ninety-two nurses participated in this survey. Only 80% (562) of the nurses had engaged in CPE activities during the past 12 months. All attendance for the various activities was below 50%. Workshops were the most popular CPE activity (345, 43.6%) and tertiary education was the most unpopular activity (10, 1.3%). The respondents did perceive the importance of future CPE activities for career development. Mandatory continuing professional education (MCPE) is a key measure to ensure that nurses upgrade their knowledge and skills; however, it is recommended that policy makers and nurse leaders in the continuing professional development unit of health service facilities plan CPE activities to meet registered nurses' (RNs) needs and not simply organizational requirements.

## 1. Introduction

Globally, all professionals have recognized the phenomenon of continuing professional education (CPE) as a primary method to enhance basic professional education regularly [1]. Globalization, technological advances, consumerism and climate changes have challenged the health care environment to ensure practice and services are contemporary. As nurses are the largest group of health care professionals globally, therefore nurses are required to participate in CPE to develop skills and competencies, and remain current in their practice. The International Council of Nurses Code of Ethics for Nurses [2] advocated that “the nurses carry personal responsibility and accountability for nursing practice, and for maintaining competence by continual learning.”

In view of the important role of CPE to nurses, many countries have implemented mandatory continuing professional education (MCPE), beginning with the State of California in 1971 [3]. Later in 1973, the American Nurses Association advocated MCPE for relicensure [4]. Currently 23 states in the United States have enforced legislation that requires nurses to participate in CPE in order to renew their license to practice [5].

Similarly, in the United Kingdom, legislation was passed and MCPE was introduced to ensure the quality of nursing [3]. Following that, nurses in Australia were required to declare that they were current. In 2009, the Nurses Board of the State of Victoria first implemented auditing nurses' CPE points and started asking for evidence of currency.

The need for MCPE is not a practice restricted to Western countries. China implemented MCPE for nurses in 1996 [6]. In Hong Kong, the Nursing Council of Hong Kong proposed a minimum requirement of 45 CPE credit points (points assigned to various recognized CPE activities; e.g., 20 points is given for attending a two-day conference) to renew their three-year practicing certificate [7].

In Malaysia, the Nursing and Midwifery Board has long recognized the importance of CPE. Initially the Board required nurses to comply with a provision in the code of professional conduct to participate in a minimum of ten hours of CPE, although this expectation was not checked with each application for practice license renewal. The Malaysian Nursing and Midwifery Board introduced MCPE in 2008. All registered nurses (RN) are now required to participate in and provide documentary evidence that they have met the minimum requirement of 25 credit points of CPE annually before renewal of licensure is granted [8]. MCPE ensures all Malaysian nurses have accepted responsibility to update their knowledge and skills, thus, ensuring their practices are safe. The responsibility for the Malaysian Midwifery and Nursing Board is to ensure that they are vigilant and adapt and include innovative CPE pedagogy in the recognized CPE activities and the preferences of the nurses, both of which may evolve. Do the RNs achieve their personal need or the institution's need? The study sought to examine if nurses believe that participating in the current accepted range of CPE activities meets their immediate and future needs. The finding from this study shall add to CPE literature from the Malaysian and the Asian country perspective in CPE for nurses.

## 2. Materials and Methods

A quantitative cross-sectional survey was designed to obtain information from the nursing population regarding CPE practice. The aim of this study was to explore the current CPE practice of Malaysian nurses and to identify the future CPE needs of this population.

### 2.1. Participants

The study population consisted of 70,000 registered nurses working in 178 Ministry of Health hospitals and 2987 community clinics. A three-stage, multistage cluster, stratified sampling method was used for this survey [9]. The first stage was to randomly select one state/territory from each of the four Peninsula Malaysia regions: Penang (Northern region), Johor (Southern region), Pahang (Eastern region), and Selangor (Central region). For the second stage all government hospitals and district health offices in each selected state were identified, from which one primary hospital, one secondary hospital, one tertiary hospital, and one health office from each state were randomly selected. The goal of the sampling method was to draw a random sample of 1000 registered nurses from west Malaysia (Peninsular Malaysia). Four states were drawn from the list of Peninsular Malaysia. Within the state, four clusters were selected, as shown in Table 1. The number of RNs from each cluster is based on the proportions listed in Table 1.

The inclusion criteria for the sample were that they must be state RNs with at least one-year working experience. The RNs selected were based on implied consent to participate in the study. Nurses who were on maternity leave, study leave, unpaid leave, or long medical leave were excluded from the study.

### 2.2. Instrument

The questionnaire was developed with considerable attention given to construct clear and unambiguous items. When items were developed, care was taken to use simple language and short sentences that were neither double-barreled nor leading. The questionnaire consist of close ended questions; likert scale was used to rank the future need of nurses on activities and topics. The present study used agreement statement “1 = not at all, 2 = a little, 3 = somewhat, 4 = moderately, 5 = a great deal” for opinion scale for importance of CPE. The questionnaire was developed based on literature search, with the main tools related to CPE [10] and consisted of three parts: part A: demographic information of the respondents; part B: concerned CPE activities; part C: importance of CPE activities and the topic of CPE. Five experts in nursing education were invited to validate the content. The internal consistency, Cronbach's alpha, of the importance of CPE activities and topic was, respectively, 0.614 and 0.779. Thirty registered nurses participated in the prestesting of the instrument.

### 2.3. Ethical Considerations

Application of ethical approval to conduct research in Malaysia was submitted to the Malaysia Economic Planning Unit, Ministry of Health in Malaysia, and University of Malaya Medical Centre. The research proposal was submitted to the Monash University Human Research Ethics Committee for ethical approval. A written cover letter was distributed to the participants clarifying the purpose of the study, together with the questionnaire. Participation was voluntary and the information provided was treated with the utmost regard for confidentially and anonymity. Since this survey was a low-risk research, consent was based on implied consent and did not require a written consent.

### 2.4. Data Collection

Data were collected in November 2008. One thousand questionnaires were distributed to RNs from 12 hospitals and 4 community health centers. By the end of January 2009, a total of 850 questionnaires had been returned; however, only 792 of the participants had correctly completed the questionnaire.

### 2.5. Data Analysis

The statistical package for social sciences (SPSS) version 16.0 for Windows was used for statistical analyses. Statistical methods included frequency, percentage, mean, and standard deviation (SD) for descriptive analysis.

## 3. Results and Discussion

### 3.1. Participant Demographics

A total of 1000 questionnaires were distributed to participants, of which 792 were returned completed giving a response rate of 79.2%.  Table 2 presents a summary of the demographic characteristics of the sample. Result shows that the majority of nurses were female (97.1%) and married (77.1%) with one or two children (52.2%). The age of nurses ranged from 23 to 56 years with a mean age of 33.89 with SD of 9.11. Examination of age groups shows a similar distribution across the three categories. A similar distribution is also noted across the four income categories, although 75% of nurses reported an income of RM 4630.00 (USD 1450) or less per month which is considered low when compared to other professions. As expected, a higher percentage of nurses were working in a tertiary hospital (55.7%) and fewer in a community health clinic (8.8%). The mean years of nursing experience was 10.2, with a range from 1 to 35 years. The highest professional qualification for the greater majority of nurses was a nursing diploma (76.0%) and only 2.3% had a nursing degree. No participants had a Master Degree or a Ph.D. qualification.

### 3.2. Participation in CPE Activities

Five hundred and sixty-two (71%) of the RNs had participated in CPE activities in the past 12 months; however only 324 (40.9%) of them had achieved the 25 or more credit points required to renew their licenses. Two hundred and thirty-eight (30.05%) had obtained less than 25 credit points, which had excluded them from relicensing.  Table 2 shows the respondents attendance at various CPE activities, categorized as never, one to two times, and more than twice. All activity attendance was below 50%. The highest attendance (345, 43.5%) was participating in workshops followed by conferences (323, 40.8%). Only 10 (1.3%) of the RNs had undertaken tertiary education. Even though the attendance rate for available CPE was low, all nurses identified the value of attending future CPE activities; the mean score for this item was above 3. The mean scores for tertiary education and advance-nursing courses were high, even though the attendance rates were low. The mean scores for conducting research and presenting papers were low compared to other activities; this is consistent with the low attendance rate.

The topics of CPE attended by respondents were consistent with their identified future needs. The nurses highlighted specialty nursing CPE, for example, renal nursing, orthopedic nursing, and community health nursing, as the most important topic.

## 4. Discussion

Only 71% of the RNs from this study had participated in CPE in the last 12 months, despite the introduction of MCPE for Malaysian RNs since 2008. Previous studies, which had been undertaken before the introduction of MCPE, showed higher rates of participation by nurses. Salim [11] conducted a survey in a teaching hospital with 68 nurses from different clinical specialty areas and found that 73.53% of the nurses had attended a CPE programme, with a mean duration of 29.1 hours per two years. Similarly, in Hong Kong, nurses reported being enthusiastic to participate in CPE, even though it is not mandated by the regulatory authority [7].

### 4.1. Types of CPE Activities

Most of the RNs in this study participated in workshops or attended conferences, followed by in-service education. These findings are consistent with previous studies that found participation in workshops, seminars, conferences, in-service programmes, and academic programmes was often the choice of CPE activities among nurses [12, 13].

In this study, participation in reading and undertaking research was low. This finding is similar to that reported by [14], which found that only 45% of the respondents indicated that they had read one or more nursing journal articles in the past two years and that only 29% of the nurses used the Internet for CPE purposes. Pathan [14] suggests that the lack of provision of nursing information, such as journals and books in clinical areas, and being discouraged from reading in the workplace has deterred nurses from engaging in this type of CPE. Lee et al. [7] reported disappointment that Hong Kong nurses were not interested in undertaking research, despite knowing the importancey of evidence-based nursing.

This study found that the rates of participation in degrees and diplomas were low compared to other activities but this is consistent with previous research [7, 10, 14, 15]. Previous studies revealed the reasons for not participating in CPE were financial constraints, family burdens, and work commitment [7, 10, 14, 15].

The nurses from this study preferred the face-to-face and structured didactic short course opportunity for CPE is often suggested by the employer. The purpose of CPE is merely to collect point to review their practice license. The fact that very few nurses attended tertiary study may be an effect of the lack of opportunity, arising from the limited number. In addition, the courses are full time and the universities are located in cities. Even though some public universities offer online courses for nurses, the cost has deterred nurses from pursuing tertiary education. The scarcity of degree-level qualifications among nurses may affect their interest in research, as they may not have the skills to undertake research and learn independently.

### 4.2. Topics of CPE

In this study, 60.7% of the nurses reported attending nursing courses in specialized areas, such as cardiothoracic nursing, wound care management, and cardio pulmonary resuscitation. This is consistent with Pathan [14] and Lee et al. [7], who found that nurses in their studies often preferred courses that they believed would enhance and advance their nursing practices and clinical work. This study found that general nursing, such as nursing process, documentation, and nursing theory, or fundamental nursing, was the second most popular topic because it included most of the in-service training courses and was usually held in the work place environment. Indirect nursing is related to communication skills or information and communication technologies (ICT) skills. The response is considered low and most of the RNs attended ICT courses to learn the basic skills. Lee et al. [7] cite poor computer literacy as the reason for this choice. Management courses were not a common choice among the nurses in this study; a finding supported by Lee et al. [7]. In their study, only 7.8% of nurses attended management courses primarily because the nurses did not cover management in their basic training and, therefore, did not value this topic. Most of nurses did not show an interest in teaching skills and research; this finding is alarming because nurses lacking teaching skills would compromise the advocacy of health promotion, and poor utilization of research among nurses may jeopardize quality of patient care.

### 4.3. Future Learning Needs

Almost all the nurses in this study positively perceived that CPE activities (641, 80.9%) and the topic (730, 92.2%) are important. The need for future CPE activities was consistent with the previous participation rate activities for in-service training, conferences, and workshops (Table 3). Most nurses want to participate in these activities in the future. Table two also shows participation of RNs in advanced nursing and degree programmes does not reflect their perceived future need; the reactively mean scores are 3.67 and 4.01, respectively, despite low attendance (12.9% and 1.3%). In other words, the nurses perceive these activities are important even though they did not participate actively. This indicates that nurses have a thirst for knowledge and are eager to upgrade their practice and knowledge.

CPE attendance was highest when the topics reflected the needs of the nurses. Specialist nursing topics, such as cardiothoracic nursing, wound care management, and cardio pulmonary resuscitation generally perceived to be advanced practice skills, were rated the most important, and this is reflective of the trend for specialization [6]. The respondents rated the mean score for teaching and research high even though very few had attended CPE activities on these. This study demonstrated that nurses understand the need to acquire teaching and research skills, which they know are vital to improve patient care. The low attendance rate could be due to inaccessibility of programmes and other deterrence factors highlighted in previous studies, such as time and financial constrains [7, 10, 15].

The inventory of future learning need for CPE serves as a yard stick to provide relevant and timely courses planned for nurses. The provision of CPE should focus on the learners' need to ensure they receive latest learning experience that they will be able to apply to improve their current practice. The staff development and in-service training unit should consider providing a more structured programme based on nurses' learning needs in cooperated collaboration in three aspects of nursing: practice, education, and research. Without proper planning and research, mandatory CPE is unlikely to deliver the anticipated development of reflective practice and critical thinking that is considered crucial to improving patient care. It is also imperative for CPE providers to examine critically the existing education approach and explore more innovative teaching methods such as e-learning and self-directed learning, taking a problem-solving approach such as problem-based learning or evidence-based nursing.

## 5. Conclusion

This study found that Malaysian nurses participation in CPE was not convincing despite of implementation of MCPE by Malaysian Nursing Board. However, the nurses acknowledge the importance and the need for future CPE. Thus, MCPE would not improve the CPE participation among nurses unless the programmes are planned and implemented based on the nurses' needs. Collaboration among the nursing leaders in every area is vital to improve our practice, and most importantly, our nursing profession.

## Figures and Tables

**Table 1:** Cluster stratified sampling.

Cluster	Tertiary hospital	Secondary hospital	Primary hospital	Health clinic	Total for each state
Proportional	1000	500	100	80	
*n*	135	65	30	20	250

**Table 2:** Demographic characteristics (*N* = 792).

Characteristic	Frequency	%
Sex		
Female	769	97.1
Male	23	2.9
Age (in years)		
≤28.0	288	36.4
29–35	252	31.8
≥36	252	31.8
Mean: 33.89 (SD = 9.11)		
Marital status		
Married	611	77.1
Single	166	21.0
Separated/divorced	6	0.8
Widow/widower	9	1.1
Number of children		
0	80	12.8
1-2	327	52.2
3-4	183	29.2
≥5	37	5.8
Household income/month (RM)		
≤2914.00	202	25.5
2915–3980	215	27.2
3981–4630	177	22.3
≥4631	198	25.0
Mean: 3941.40 (SD = 1633.38)		
Working institution		
Tertiary hospital	441	55.7
Secondary hospital	164	20.7
Primary hospital	117	14.8
Health clinic	70	8.8
Years of service		
≤3	203	25.6
4–8	234	29.5
9–14	164	20.7
≥15	191	24.2
Mean: 10.16 (SD = 8.54)		
Professional education		
Diploma in nursing	602	76.0
Advance diploma in nursing	172	21.7
Degree in nursing	18	2.3

**Table 3:** CPE practice (*N* = 792).

CPE practices	Respondents' attendance (*N* = 792)			Perceived need for future CPE activities and topics to respondents' professional/career development
	Never	1-2	>2	
	*n* (%)	*n* (%)	*n* (%)	Mean
Type of activity				
In-service education	501 (63.3)	96 (12.1)	195 (24.6)	4.10
Advanced nursing courses	690 (87.1)	102 (12.9)	0	4.01
Workshops	447 (56.4)	290 (36.6)	55 (6.9)	3.83
Orientation programme	591 (74.6)	189 (23.9)	12 (1.5)	3.80
Conference	469 (59.2)	261 (33.0)	62 (7.8)	3.67
Tertiary education	782 (98.7)	10 (1.3)	0	3.67
Reading nursing journals	649 (81.9)	82 (10.4)	61 (7.7)	3.61
Carried out research	756 (95.5)	36 (4.5)	0	3.21
Presented at a conference	719 (90.8)	63 (8.0)	10 (1.3)	3.19
Topic of CPE				
Specialty nursing	311 (39.3)	317 (40)	164 (20.7)	4.90
General nursing	396 (50)	192 (24.2)	204 (25.8)	4.20
Indirect nursing	544 (68.7)	150 (18.9)	98 (12.4)	4.00
Management	658 (83.1)	109 (13.8)	25 (3.2)	4.00
Teaching	728 (91.9)	63 (8.0)	1 (0.1)	3.90
Research	641 (80.9)	141 (17.8)	10 (1.3)	3.50
